# Downregulation of Swine Leukocyte Antigen Expression Decreases the Strength of Xenogeneic Immune Responses towards Renal Proximal Tubular Epithelial Cells

**DOI:** 10.3390/ijms241612711

**Published:** 2023-08-12

**Authors:** Katharina Schmalkuche, Reinhard Schwinzer, Nadine Wenzel, Emilio Valdivia, Björn Petersen, Rainer Blasczyk, Constanca Figueiredo

**Affiliations:** 1 Institute of Transfusion Medicine and Transplant Engineering, Hannover Medical School, Carl-Neuberg-Str. 1, 30625 Hanover, Germany; 2 Transregional Collaborative Research Centre 127, Hannover Medical School, Carl-Neuberg-Str. 1, 30625 Hanover, Germany; 3 Transplantation Laboratory, Clinic for General, Visceral and Transplantation-Surgery, Hannover Medical School, Carl-Neuberg-Str. 1, 30625 Hanover, Germany; 4 Institute of Farm Animal Genetics, Höltystr. 10, 31535 Neustadt am Rübenberge, Germany

**Keywords:** SLA, RNAi, kidney, xenotransplantation, immortalization

## Abstract

Xenotransplantation reemerged as a promising alternative to conventional transplantation enlarging the available organ pool. However, success of xenotransplantation depends on the design and selection of specific genetic modifications and on the development of robust assays allowing for a precise assessment of tissue-specific immune responses. Nevertheless, cell-based assays are often compromised by low proliferative capacity of primary cells. Proximal tubular epithelial cells (PTECs) play a crucial role in kidney function. Here, we generated immortalized PTECs (imPTECs) by overexpression of simian virus 40 T large antigen. ImPTECs not only showed typical morphology and phenotype, but, in contrast to primary PTECs, they maintained steady cell cycling rates and functionality. Furthermore, swine leukocyte antigen (SLA) class I and class II transcript levels were reduced by up to 85% after transduction with lentiviral vectors encoding for short hairpin RNAs targeting β2-microglobulin and the class II transactivator. This contributed to reducing xenogeneic T-cell cytotoxicity (*p* < 0.01) and decreasing secretion of pro-inflammatory cytokines such as IL-6 and IFN-γ. This study showed the feasibility of generating highly proliferative PTECs and the development of tissue-specific immunomonitoring assays. Silencing SLA expression on PTECs was demonstrated to be an effective strategy to prevent xenogeneic cellular immune responses and may strongly support graft survival after xenotransplantation.

## 1. Introduction

The kidney regulates vital processes such as the regulation of blood pressure, osmoregulation, or the maintenance of the acid-base balance [1]. Thus, patients suffering from end-stage kidney disease rely on dialysis or highly desirable kidney transplantation as the most effective treatment option. However, both scarcity of organs and genetic disparities between donor and recipient, mainly based on human leukocyte antigen loci, often lead to long waiting times until kidney transplantation [2,3,4,5]. Currently, a mortality rate of 15% is observed in patients waiting on average 3.9 years for a suitable organ [6]. Kidney xenotransplantation may represent a promising alternative to circumvent the shortage of human organs. Nevertheless, genetic differences between pigs and humans trigger severe immune responses contributing to graft failure and rejection. Recently, life-sustaining pig kidney xenografts were proven to survive for approximately 500 days and orthotropic heart xenografts for up to 200 days in non-human primates [7]. In addition, as a remarkable milestone, the first genetically modified porcine heart and kidney were transplanted into human; however, the patients survived only weeks to months after xenotransplantation [8,9].

Hence, the development of robust assays to predict the strength of xenogeneic immune responses targeting kidney cells is crucial to support the development of kidney xenotransplantation. Proximal tubular epithelial cells (PTECs) are extensively involved in renal clearance and tissue regeneration after injury or inflammation. They are essential for the recovery of important metabolites by active transport as well [10,11,12]. Besides the endothelium, PTECs show relevant immunoregulatory functions during immune rejection after kidney transplantation [13]. Therefore, in this study, we focused on evaluating the feasibility of generating and using immortalized PTECs (imPTECs) to investigate the impact of specific genetic modifications on the strength of the immune response. The development of specific in vitro assays may become central in the evaluation of xenograft survival after transplantation. However, freshly isolated PTEC proliferation capability is limited, which means that they are unsuitable for routine testing or require the use of additional animals to obtain sufficient cellular material. Hence, the establishment of PTEC lines would facilitate the development of in vitro assays in different diagnostic and research areas to evaluate the feasibility and efficiency of new therapeutic strategies to reduce and optimize the need for experimental animals. 

## 2. Results

### 2.1. Immortalized PTECs Maintain Typical Cell Morphology 

**Figure 1:**
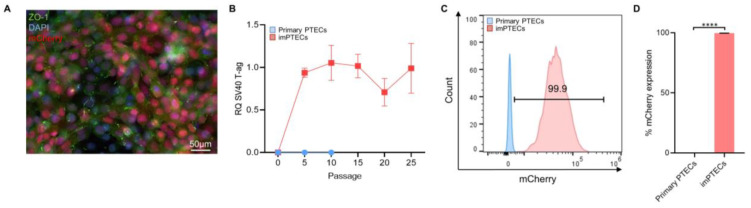
Immortalization of proximal tubular epithelial cells (PTECs) by transduction with simian virus 40 (*SV40)* large T-antigen (ag)-encoding lentiviral vector. (**A**) Representative picture of immortalized PTECs (imPTECs) expressing mCherry. PTEC nuclei were stained with DAPI (blue) and the tight junction protein-1 (ZO-1) marker is displayed in green. (**B**) Relative quantification of SV40 large T-ag expression on primary and imPTECs (*n* = 3). (**C**) Representative histogram of mCherry fluorescence of primary and imPTECs. (**D**) Comparison of primary and imPTEC mCherry expression (*n* = 3). Statistical significance was evaluated using unpaired *t*-test (**** *p* < 0.0001).

After immortalization of porcine PTECs with lentiviral vectors harboring the complete simian virus 40 (*SV40)* large T-antigen (ag) sequence, stable high levels of SV40 large T-ag transcripts were detected at several passages (passage 5: 0.94 ± 0.05; passage 10: 1.05 ± 0.17; passage 15: 1.02 ± 0.11; passage 20: 0.71 ± 0.13; passage 25: 0.99 ± 0.24) (Figure 1A,B). mCherry-positive populations were isolated by cell sorting with a purity of 99.73 ± 0.24% (*p* < 0.0001) and expanded to generate cell lines (Figure 1C,D). 

**Figure 2:**
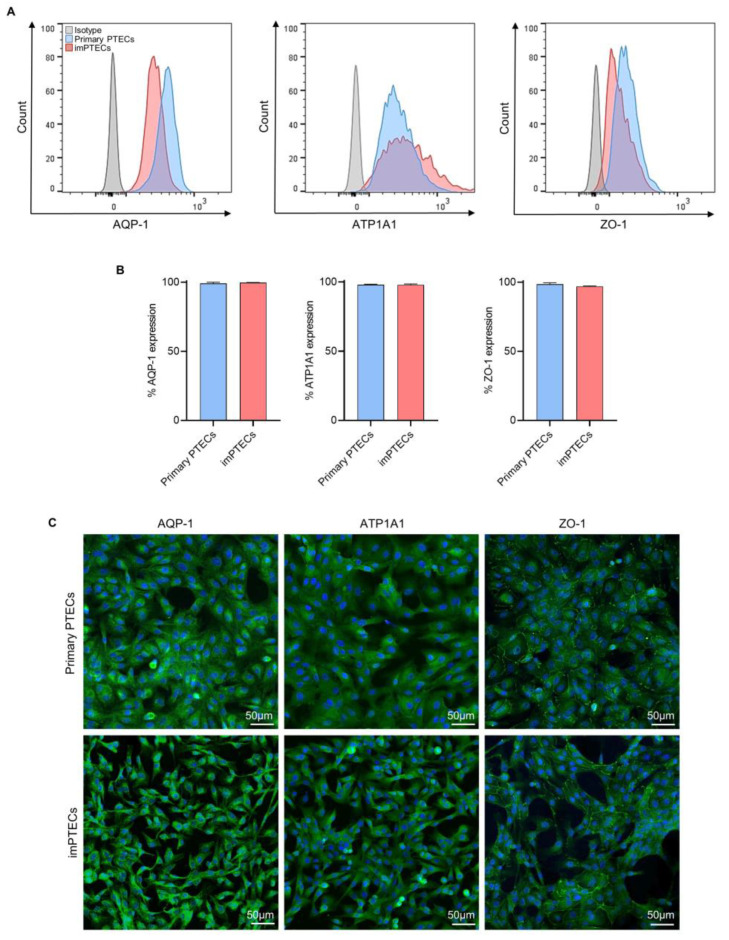
Phenotypical characterization of primary and immortalized PTECs. (**A**) Representative overlay histograms of aquaporin-1 (AQP1), sodium/potassium-transporting ATPase subunit alpha-1 (ATP1A1), and ZO-1 expression on primary (passage 2) and immortalized cells (passage 10). (**B**) Graphs show mean expression (*n* = 3) of AQP1, ATP1A1, and ZO-1. (**C**) Immunofluorescence analysis of specific proteins (AQP1, ATP1A1, and ZO-1) expressed on primary (passage 2) and imPTEC (passage 10) lines. Marker expression is represented in green and DAPI in blue. Statistical significance was evaluated using unpaired *t*-test.

The expression of the PTEC-specific markers aquaporin-1 (AQP-1), sodium/potassium-transporting ATPase subunit alpha-1 (ATP1A1), and tight junction protein-1 (ZO-1) showed no significant differences between primary and immortalized PTECs (Figure 2A–C). Protein expression frequencies of AQP1, ATP1A1, and ZO-1 on primary PTECs were 99.10 ± 0.78%, 98.10 ± 0.29%, 98.60 ± 0.85% and 99.77 ± 0.13%, 97.93 ± 2.33%, 96.98 ± 1.67% on imPTECs, respectively (Figure 2B). These data suggest that transduction and cell immortalization do not affect PTEC phenotype significantly.

### 2.2. ImPTECs Exhibit Altered Growth Dynamics

**Figure 3:**
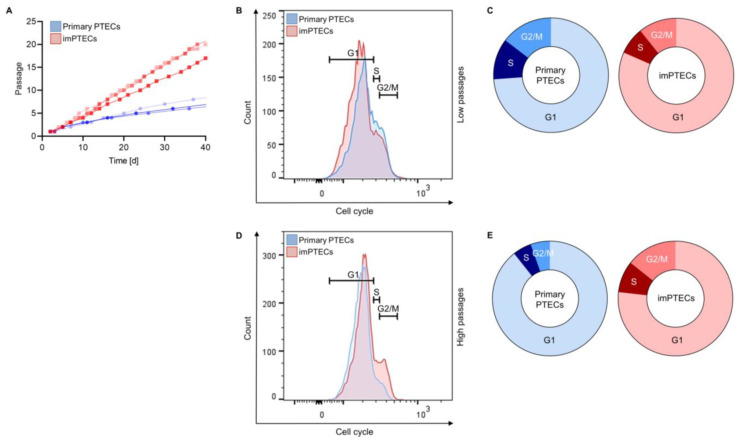
ImPTECs exhibit altered growth dynamics. (**A**) Doubling population analysis of primary and imPTECs (*n* = 3). Representative overlay histograms of cell cycling analyses of primary and imPTECs at low passages (passage 2) (**B**) and high passages (passage 10) (**D**). Proportion of primary (**C**) and imPTECs (**E**) in each phase cycle stage (*n* = 3). Statistical significance was evaluated using one-way ANOVA.

Immediately after passage 3, imPTEC proliferation rate was significantly (*p* < 0.01) increased compared to primary PTECs. The population doubling time of primary PTECs equaled 11.0 ± 0.82 days in comparison to 6.67 ± 0.47 days of imPTECs. Also, after passage 6, significantly higher proliferation rates (*p* < 0.01) were observed comparing imPTECs with primary PTECs. Primary PTECs required 30.67 ± 4.99 days to increase their number by 12-fold of the initial number. In contrast, imPTECs showed a 12-fold cell number increase 12.67 ± 1.25 days after transduction. In fact, imPTEC population doubled on average within 2 days and remained unaltered by passage 20, in contrast to the primary PTECs, where the doubling rate decreased with a high number of passages (Figure 3A).

Cell cycle analysis of PTECs at low passage numbers revealed frequencies of 73.85 ± 0.80% of primary PTECs in G1 in comparison to 80.59 ± 2.04% imPTECs shortly after transduction (passage 2). Higher frequencies of primary cells were localized in the S phase of the cell cycle in comparison to imPTECs (11.55 ± 0.05% vs. 7.67 ± 0.21%, *p* < 0.0001). Similarly, 14.60 ± 0.80% of primary PTECs and 10.73 ± 0.60% of imPTECs (*p* < 0.01) were observed in the G2/M phase of the cell cycle (Figure 3B,C). Remarkably, as expected, at higher passages (passage 10), an increased frequency of imPTECs was detected in the G2 phase and lower frequencies of cells in the G1 phase. Compared to the primary cells (G1 phase: 89.32 ± 0.57%; S phase: 5.27 ± 0.25%; G2 phase: 5.40 ± 0.39%), imPTECs were represented by 76.79 ± 0.72% (*p* < 0.0001) in the G1 phase, 8.902 ± 0.30% (*p* < 0.0001) in the S phase, and 14.30 ± 0.62% (*p* < 0.0001) in the G2/M phase (Figure 3D,E). These results indicate that the majority of primary PTECs remain at higher passages in the G1 phase, while cell cycle phases of immortalized cells remain almost unaffected at later passages.

### 2.3. Immortalization Does Not Affect Cell Function

Functional PTECs exhibit the ability of albumin endocytosis by reabsorbing albumin into the proximal tubule [14]. At low passages (passage 2), primary and imPTECs cultured in presence of FITC-conjugated albumin showed comparable mean fluorescence intensity (MFI) levels of 4099.67 ± 279.06 and 4337.67 ± 477.40, respectively, indicating a similar capacity to uptake albumin. In contrast, at high passages (passage 20), primary PTECs reached MFI of 3471.00 ± 340.85 and showed a significantly (*p* < 0.01) decreased ability to uptake albumin in comparison to imPTECs, where functionality remained comparable to lower passages (MFI: 4528.67 ± 131.87). These data suggest that imPTECs maintain typical cell function after immortalization and, in contrast to primary cells, maintain it even at higher passages.

**Figure 4:**
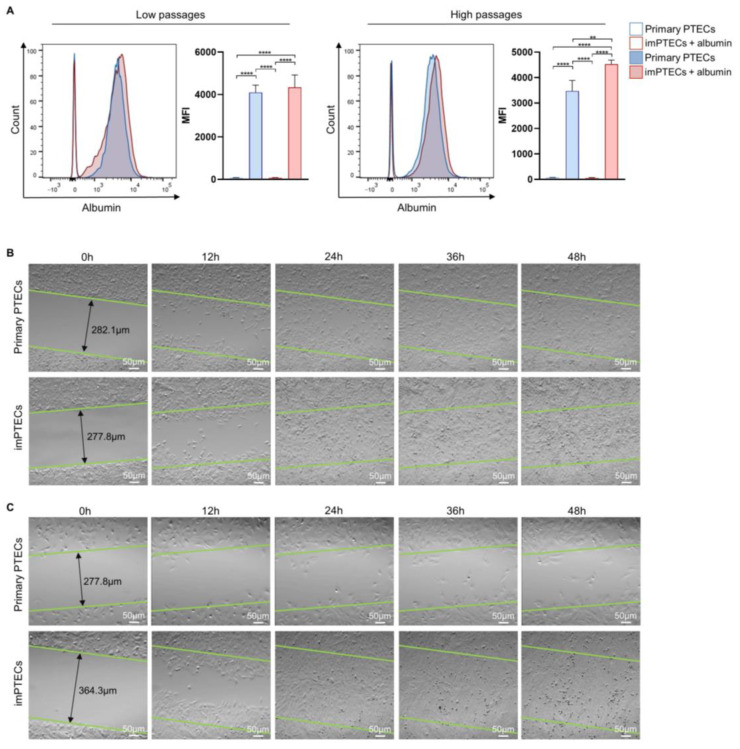
Functional capacity of imPTECs remains constant even at high passages. (**A**) Albumin uptake in primary and imPTECs analyzed by flow cytometric analyses after incubation with BSA-FITC. Representative histogram plots show albumin uptake in low and high passages in comparison to primary and imPTECs. Statistical significance was evaluated using one-way ANOVA (** *p* < 0.01; **** *p* < 0.0001). (**B**,**C**) The migration capacity of PTECs was evaluated in scratch assays. Pictures display time-lapse sequences of the capacity of primary and imPTECs to close gaps over 48 h. Images were collected every 12 h. Representative pictures of PTECs at low passages (passage 2) (**B**) and at high passages (passage 10) (**C**).

Epithelial cells play a crucial function during wound-healing processes, which are of great importance concerning transplantation. In particular, proliferation and migration represent important mechanisms during tissue repair [15]. Therefore, the migratory ability of PTECs was used to evaluate the functionality of imPTEC lines in scratch assays. After 24 h, imPTECs (passage 2) completely closed the scratch, whereas primary cells migrated slowly and only overgrew the gap after 36 h (Figure 4B). This difference became even more obvious when analyzing the migration capability at higher passages (passage 10). Primary PTECs at passage 10 exhibited significantly lower migration and proliferation rates compared to passage 2 and were not capable of closing the scratch even after 48 h, whereas immortalized PTECs formed a confluent monolayer after 24 h (Figure 4C). These data show that imPTECs are not limited in their functionality even at high passages.

### 2.4. Silencing SLA Class I and Class II Expression by Genetic Engineering of imPTECs

The use of xenogeneic organs as an alternative to increasing the pool of organs suitable for transplantation is associated with the necessity of genetic modification to overcome immunological barriers posed by phylogenetic discrepancies between pigs and humans. One of the strategies proposed to reduce xenograft immunogenicity is the downregulation of swine leukocyte antigen (SLA) class I and class II expression [16]. A robust silencing of SLA class I and class II expression may decrease the strength of allo- or xenogeneic immune responses by generating an immunologically invisible status of tissues and organs supporting graft survival [17,18,19]. The use of PTECs isolated from genetically modified pigs or in vitro genetically modified PTECs may contribute to evaluate the effect of specific genetic modifications in human immune response towards porcine-derived kidneys and to monitor the status and strength of xenogeneic immune response after transplantation.

**Figure 5:**
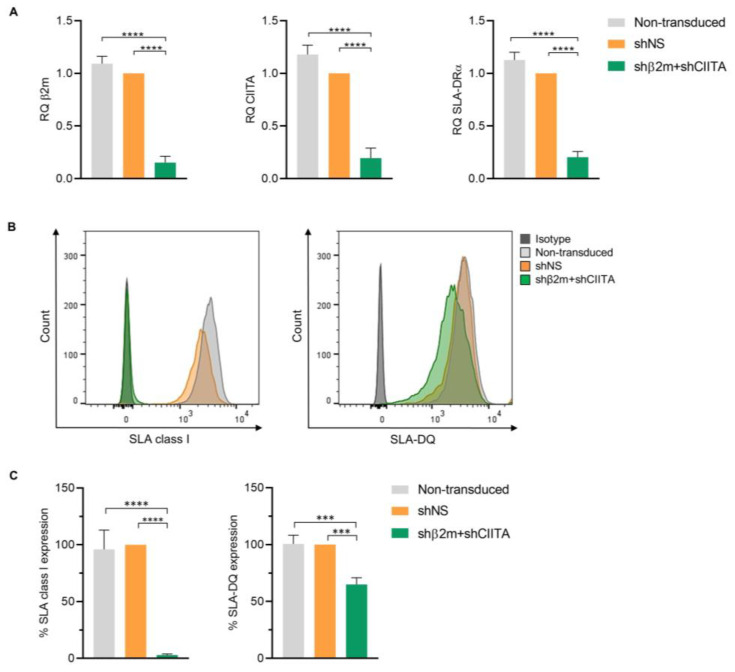
Silencing swine leukocyte antigen (SLA) class I and class II expression on imPTECs. (**A**) Relative quantification (RQ) of β2-microglobulin (β2m), class II transactivator (CIITA), and SLA-DRα transcript levels detected in non-transduced, non-silenced (shNS), and silenced (shβ2m + shCIITA) imPTECs (*n* = 3). (**B**) Representative histogram plots of SLA class I and SLA-DQ expression on non-transduced, non-silenced (shNS), and silenced (shβ2m + shCIITA) imPTECs. (**C**) Mean and standard deviation of SLA class I and class II expression levels on non-transduced, non-silenced (shNS), and silenced (shβ2m + shCIITA) imPTECs (*n* = 3). Statistical significance was evaluated using one-way ANOVA (*** *p* < 0.001; **** *p* < 0.0001).

Immortalized PTECs, genetically engineered using lentiviral vectors encoding for short hairpin (sh) RNAs targeting beta-2-microglobulin (β2m) and class II transactivator (CIITA), showed a silencing effect on β2m, CIITA, and SLA-DRα mRNA transcript levels of 84.66 ± 0.049% (*p* < 0.0001), 80.47 ± 0.076% (*p* < 0.0001), and 79.58 ± 0.044% (*p* < 0.0001), respectively, compared to cells transduced with lentiviral particles encoding for non-specific shRNA (shNS) (Figure 5A). Correspondingly, in comparison to SLA-expressing (shNS) imPTECs, the silencing effect on β2m and CIITA transcript levels was translated into a downregulation on SLA class I and class II surface expression of 97.02 ± 0.70% (*p* < 0.0001) and 34.96 ± 4.81% (*p* < 0.001), respectively (Figure 5B,C). These data demonstrate the feasibility of regulating SLA class I and class II transcript and protein expression after vector-mediated shRNA delivery to imPTECs. 

### 2.5. SLA-Silenced PTECs Induce Weaker Xenogeneic T-Cell Responses

**Figure 6:**
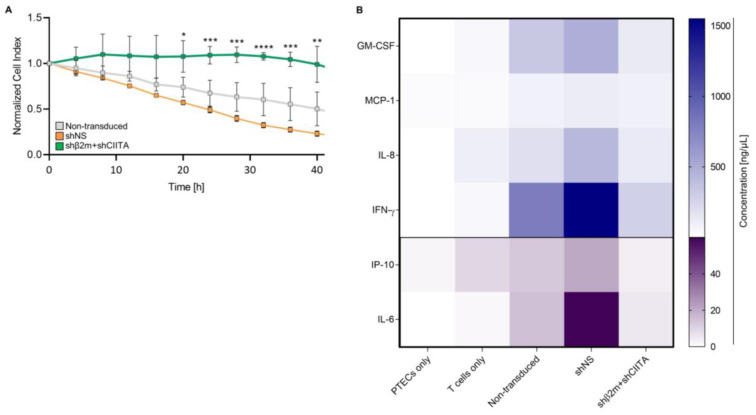
SLA class I and class II silenced imPTECs are protected against xenogenic T-cell responses. (**A**) Normalized cell index of non-transduced, non-silenced (shNS), and SLA-silenced (shβ2m + shCIITA) imPTECs co-cultured with xenoreactive T cells (ratio 1:5) for 6 days. Statistical significance was evaluated using two-way ANOVA (* *p* < 0.05; ** *p* < 0.01; *** *p* < 0.001; **** *p* < 0.0001). (**B**) Heat map represents xenoreactive T-cell cytokine secretion of granulocyte–macrophage colony-stimulating factor (GM-CSF), interferon-gamma (IFN-γ), monocyte chemoattractant protein 1 (MCP-1), interleukin 6 (IL-6), interleukin 8 (IL-8), and interferon gamma-induced protein 10 (IP-10). Cytokine concentrations were detected after incubation of non-transduced, non-silenced, and SLA-silenced imPTECs with human T cells and culturing PTECs or T cells without human T cells.

T-cell-mediated cytotoxicity is a crucial evaluation parameter of the strength of xenogeneic immune responses. Even a reduced number of xenoreactive T cells can potentially initiate a strong cellular response followed by organ rejection [20,21]. Our results showed that the T-cell cytotoxic activity is decreased in the presence of SLA I and II silenced PTECs compared to the SLA expressing (non-transduced or shNS) PTECs. SLA class I and class II silenced cells indicated higher cell survival rates than control groups after the exposition to xenoreactive T cells. The normalized cell index of SLA-downregulated PTECs was significantly (e.g., 32 h: *p* < 0.0001) increased in comparison to SLA-expressing (shNS) PTECs. For instance, after 32 h, the mean normalized cell index of the SLA class I and class II silenced PTECs (shβ2m + shCIITA) was 3.33-fold increased and reached their maximum cell index after 28 h. Remarkably, after 48 h, the survival rate of silenced PTECs (shβ2m + shCIITA) was still increased compared to the control groups (Figure 6A). These data demonstrate that the use of imPTECs is suitable for accessing xenoreactive T-cell cytotoxicity and suggest that silencing SLA expression protects PTECs against T-cell-mediated lysis.

Cytokines are important immunomodulators in the context of xenotransplantation providing information on the status of immune responses after transplantation [22]. Thus, we characterized the cytokine secretion profile of xenoreactive T cells after exposure to fully SLA-expressing or SLA-silenced imPTECs in vitro. Levels of all measured cytokines were decreased in culture supernatants of SLA-silenced (shβ2m + shCIITA) PTECs exposed to xenoreactive T cells compared to those from shNS-transduced PTECs (Figure 6B). Remarkably, cytokine secretion of IFN-γ (*p* < 0.0001) and IL-6 (*p* < 0.01) were significantly reduced to 280.2 pg/mL and 5.52 pg/mL in supernatants where shβ2m + shCIITA-transduced PTECs were used as a target in comparison to those from cultures using shNS expressing PTECs (IFN-γ 1550.35 pg/mL; IL-6 59.84 pg/mL). These results suggest that silencing SLA expression on PTECs might contribute to decreasing the strength of xenogeneic immune responses targeting the pig kidney. In addition, these assays indicate that the generation of imPTECs represents a robust strategy to generate sufficient material to reproducibly monitor the strength of the immune response.

### 2.6. Silencing SLA Class I Expression on ImPTECs Does Not Trigger NK Cell Cytotoxicity

NK cell activity may play a relevant role in the survival of xenografts. NK cell cytotoxic activity might be activated due to the absence of inhibitory ligands such as MHC class I molecules or upon the binding of xenograft-specific antibodies [23]. Here, we investigated whether silencing the expression of SLA class I on renal cells and in particular on PTECs increases the susceptibility to NK cell cytotoxicity. Hence, generated immortalized SLA-silenced porcine PTECs were co-cultured with human NK cells. Since resting NK cells retain CD107a predominantly in their intracytoplasmic granular membranes, detection of CD107a on the cell surface is associated with cytotoxic activation and degranulation [22,24].

**Figure 7:**
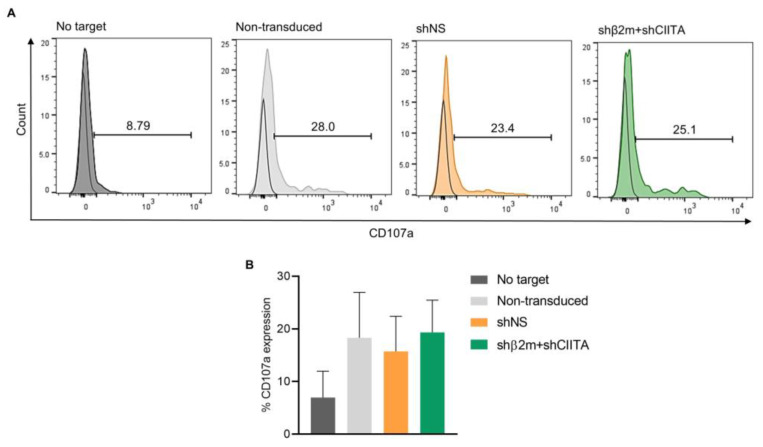
Silencing SLA class I expression on imPTECs does not trigger NK cell cytotoxicity. (**A**) Representative histogram plots of CD107a expression after NK cell exposure to no target (basal levels), non-transduced, non-silenced (shNS), and SLA-silenced (shβ2m + shCIITA) PTECs. (**B**) Mean and standard deviations of percentages of CD107a expression levels. Statistical significance was evaluated using one-way ANOVA.

Interestingly, reduced SLA expression on silenced PTECs did not increase the NK cell surface expression of CD107a (Figure 7A,B). Expression levels of CD107a on the NK cell surface were comparable upon exposure to non-transduced (18.30 ± 7.06%), non-silenced (shNS, 15.73 ± 5.43%), or SLA-silenced (shβ2m + CIITA, 19.33 ± 5.00%) PTEC targets. Altogether, CD107a expression levels showed no significant differences between SLA-silenced PTECs in comparison to the SLA-expressing control groups. These results suggest that silencing of SLA class I expression does not induce xenogeneic NK cell activation targeting renal epithelial cells.

## 3. Discussion

The use of porcine organs for xenotransplantation may enlarge the organ pool available to treat patients suffering from ESRD [25]. However, histocompatibility hurdles between the porcine organs and the human recipients need to be minimized or circumvented to support the success of this therapy [7]. Remarkable progress in the field of xenotransplantation has been achieved, generating enormous advances [8,9]. In particular, the generation of genetically engineered pigs leads to significant improvements in xenograft survival [26]. Nevertheless, the immunological discrepancies between both species still pose a relevant hurdle by triggering immune rejection. In addition, based on phenotypic properties, specific cell types constituting the different organs may exhibit various levels of immunogenicity and are capable of triggering xenogeneic immune responses with different strengths [7]. Therefore, it is highly desirable to develop robust in vitro assays to investigate the feasibility and potency of specific genetic modifications on porcine cells in the regulation of xenogeneic immune responses. Furthermore, in vitro assays may allow the prediction of xenogeneic immune response targeting a specific organ and support the development of adequate immunosuppression regimens. Precise in vitro prediction of human immune responses may also allow the reduction in the number of non-human primates used in pre-clinical studies [27]. As the kidney is one of the most transplanted organs, we focused on the generation of renal epithelial cell lines that could be used to develop immunological assays as well as to serve as the basis of biotechnological platforms to evaluate the effect of specific genetic modifications and their impact on the strength of the human xenogeneic responses [28]. Here, we generated three PTEC lines and focused on their role in immune responses that may occur after kidney xenotransplantation. 

SV40 large T-ag binds protein products of *p53* and retinoblastoma genes and inactivates *SEN6* [29]. The inactivation of Rb-proteins leads to the activation of transcription factor E2F, which initiates S-progression and promotes immortalization and proliferation [30]. It has already been reported that the rate of cell proliferation is related to the frequency of cells in different cell cycle phases. Flow cytometry analyses of the S-phase was shown to be most useful to assessing the cell proliferative capacity [31]. In our study, SV40 large T-ag supported a steady proliferative capacity of PTECs without significant alteration of morphology and phenotype. Recently, however, other strategies for generating immortalized cells have been described. For instance, the expression of the human telomerase reverse transcriptase (hTERT) or adenovirus early region 1A (*E1A*) gene has been widely used to generate immortalized cell lines [32]. 

The ability of PTECs to uptake albumin through endocytosis is commonly used to assess their functional capacity [14,33]. In this study, we demonstrated that immortalization may support the maintenance of PTEC functional capacity. This is consistent with the data obtained from the cell cycle analyses, which also indicated that imPTECs do not lose their characteristics even after multiple passages.

Furthermore, to evaluate whether *SV40* large T-ag expression affects PTEC functionality, we tested its capacity to migrate. Previous studies have shown that epithelial cells, especially after injury, exhibit a high capacity for tissue repair and regeneration compared to their healthy state in which they are mitotically quiescent [11,12]. In the kidney, this repair function is not provided by identifiable stem cell populations as compared to organs with high rates of cell turnover. Instead, the resident tubular cells dedifferentiate into potential progenitors for self-repair and proliferate after injury. Consequently, through increasing the mitotic activity of progenitor cells, they participate in wound-healing processes after transplantation [15,34].

Generated imPTEC lines showed an increased migratory capacity even at higher passages in comparison to primary PTECs, suggesting that they maintain their cell functionality despite immortalization. The maintenance of renal PTEC typical phenotype and properties demonstrates their suitability for the development of in vitro testing assays.

Primarily, we generated those cell lines to characterize and predict potential human xenogeneic immune responses toward kidney tubular epithelial cells. Due to the genetic discrepancy between the different species, rejection often occurs. This problem has been reduced by the development of genetically modified pig kidneys. In addition to the prevention of hyperacute rejection by alpha-1,3-galactosyltransferase knockout, there is a multitude of candidate targets that might contribute to minimizing the immune response against the xenograft [35]. Immortalized PTECs might play a relevant role in the evaluation of the potency of newly developed genetic modifications and allow the selection of the most effective. 

In previous studies, we and others have shown the potential of silencing MHC expression to prevent immune responses. Recently, MHC knockout pigs were generated to provide organs with the capacity to evade immune responses after organ xenotransplantation [17,36]. In this study, we evaluated the effect of silencing SLA class I and class II expression on imPTECs, as an in vitro model to predict the strength of xenogeneic immune responses. In an allogeneic setting, we have previously demonstrated the possibility of stably silencing the expression of MHC class I and II on complete organs such as the rat kidney, limb, or pig lung. These studies showed the possibility to silence MHC expression on complex 3D structures consisting of a variety of cell types and with different immunogenic potentials [16,18,19]. Despite the value of the possibility to evaluate the strength of immune responses towards a specific organ cell type, which may contribute to further clarifying mechanisms of rejection in xenotransplantation, the use of more complex systems may contribute to assessing such responses in a more physiological setting. Two- and three-dimensional cell culture models such as spheroids or organoids may allow a more accurate evaluation of specific responses as, due to their complex structure and multitype cell composition, they may better represent native tissues and organs [37]. Therefore, in a further step of this study, 2D and 3D cultures of renal spheroids and organoids consisting of MHC-silenced renal endothelial and epithelial cells will be generated to investigate xenogeneic immune responses. 

In the allogeneic transplantation model, silencing MHC expression on different cell types, tissues, and organs was shown to be feasible and contributed to decreasing the susceptibility to antibody-mediated cellular and complement-dependent cytotoxicity. Furthermore, silencing MHC expression was shown to induce weaker T-cell allogeneic immune responses and contribute to the significantly lower secretion of pro-inflammatory cytokines of IL-6 and IFN-γ [18,19,38,39]. IL-6 is involved in innate immune responses as well as in adaptive immunity. The excessive release of IL-6 correlates with the activation of T helper cells and the inhibition of regulatory T cells, which may support an inflammatory response or autoimmune diseases after transplantation [40]. IFN-γ may directly act on kidney IFN-γ receptors supporting immune cell infiltration of the graft and amplification of the immune response. In addition, IFN-γ contributes to the upregulation of MHC on the graft [41]. Here, we could demonstrate that SLA-silenced PTECs could escape the specific T-cell cytotoxic attack and they also induced a reduced T-cell cytokine secretion. In accordance with the results achieved in this study using renal cells, Carvalho-Oliveira et al. also showed that downregulation of SLA expression on pancreatic beta-cells significantly decreases xenogeneic T-cell immune responses in vitro [42]. Hence, silencing SLA expression might represent a promising strategy to protect xenografts against T-cell responses.

Furthermore, xenogeneic NK cell responses can be induced by the engagement of DSA bound to the target cell surface, the lack of self-MHC molecules, and the overexpression of activating ligands (e.g., NKG2D) or FASL–FAS interaction. Target cell recognition induces NK cell cytotoxic attack by the release of lytic granules containing cytotoxic molecules such as perforins and granzymes as well as the secretion of pro-inflammatory cytokines (e.g., IFN-γ, TNF-α) [23,43]. The lack of MHC class I molecules on the surface of target cells induces the activation of NK cells and cytotoxic activity [44]. Here, the genetic modification of imPTECs demonstrated that silencing SLA class I and class II expression does not cause an upregulation of NK cell degranulation, suggesting that the remaining SLA class I expression is sufficient to prevent NK cell activation. 

In conclusion, we demonstrated that imPTECs are suitable for developing immune assays aiming at the evaluation of the strength of the human immune response against porcine PTECs. We showed that transduction of primary PTECs with *SV40* large T-ag is an efficient strategy to increase their lifespan without significantly compromising their phenotype or function and may pave the development of robust immunomonitoring assays. Remarkably, silencing SLA class I and class II expression was shown to reduce porcine PTEC immunogenicity, leading to weaker xenogeneic immune responses. Hence, silencing SLA expression on the porcine kidney may represent a promising option to support graft survival in kidney xenotransplantation.

## 4. Material and Methods

### 4.1. Isolation of PTECs

Animal experiments were approved by the supervisory authority (LAVES—Niedersächsisches Landesamt für Verbraucherschutz und Lebensmittelsicherheit) according to the recommendation of their Ethics Committee (LAVES, AZ 33.19-42502-04-16/2343) and conducted in compliance with the ARRIVE guidelines, the German animal welfare law, the German guidelines for animal welfare, and EU Directive 2010/63/EU. All experiments were performed in accordance with relevant guidelines and regulations.

Landrace pig kidneys were preserved on static cold storage since retrieval and during transport to the laboratory. Kidney biopsies were collected from the renal cortex and incubated for 1 h at 37 °C with collagenase type II (Gibco, Life Technologies Corporation, New York City, NY, USA). After biopsy digestion, the cell suspension was centrifuged, and the cell pellet was resuspended in CnT-Prime Epithelial Cell Culture Medium (CnT-Prime Medium) (CELLnTEC ADVANCED CELL SYSTEMS AG, Bern, Switzerland) supplemented with 2% fetal bovine serum (FCS) (Sigma-Aldrich, St. Loius, MO, USA), penicillin–streptomycin 2% (C.C.Pro, Oberdorla, Germany) and seeded onto 6-well plates. The medium was replaced every second day.

### 4.2. Phenotypical Characterization of PTECs

Cell phenotyping was performed by analyzing typical PTEC markers. After performing intracellular staining using the IntraPrep Permeabilization Kit (BD Biosciences, Franklin Lakes, NJ, USA), primary PTECs (passage 2) and imPTECs (passage 10) were incubated with primary antibodies anti-(AQP-1) monoclonal antibody (OTI2D10; Invitrogen, Carlsbad, CA, USA) followed by FITC-conjugated secondary antibody (RMG1-1; BioLegend, San Diego, CA, USA) staining or anti-(ATP1A1) recombinant rabbit monoclonal antibody (ST0533; Invitrogen) and anti-(ZO-1) polyclonal antibody (Invitrogen) followed by FITC-conjugated polyclonal antibody (BioLegend) staining. Data acquisition was performed with a BD FACSCanto™ II Clinical Flow Cytometer System (BD Biosciences), and results were analyzed using FlowJo software v10.6.2 (BD Biosciences).

PTECs were seeded into 8-well tissue culture chambers (Sarstedt, Nümbrecht, Germany). After reaching confluence, cells were fixed in 4% paraformaldehyde for 1 h. Cell permeabilization was performed using 0.1% Triton X-100 (Sigma-Aldrich) and blocked with 1% bovine serum albumin (Sigma-Aldrich) for 1 h. Primary staining was performed for 1 h using anti-(AQP-1) monoclonal antibody (OTI2D10; Invitrogen) followed by FITC-conjugated secondary antibody (RMG1-1; BioLegend) or anti-(ATP1A1) recombinant rabbit monoclonal antibody (ST0533; Invitrogen) and anti-(ZO-1) polyclonal antibody (Invitrogen) followed by FITC-conjugated polyclonal antibody (BioLegend). Cell nuclei were visualized using IS Mountain medium containing DAPI (Dianova, Hamburg, Germany) and incubated overnight at 4 °C. Images were performed using a Keyence BZ-8100E microscope (KEYENCE, Neu-Isenburg, Germany).

### 4.3. Lentiviral Vector Production

Immortalization of PTECs was performed using lentiviral vectors encoding for the *SV40* large T-ag and mCherry sequence as a reporter gene. Additionally, lentiviral vectors carrying the sequences for shRNAs targeting β2-microglobulin (β2m) and the class II transactivator (CIITA), as well as sequences for a control vector encoding for non-sense shRNA (shNS) were produced as previously described [16]. Briefly, HEK293T cells were cultured in HYPERFlask Cell Culture Vessels (Corning, New York, NY, USA) in Dulbecco’s modified Eagle´s medium supplemented with 10% FCS (Sigma-Aldrich), 2% penicillin–streptomycin (C.C.Pro), and 1% glutamine (PAN-Biotech GmbH, Aidenbach, Germany). These conditions were used for the production of all three vectors. HEK293T cells were cotransfected with the lentiviral packaging plasmid psPAX2, the VSV-G envelope plasmid pMD2.G, and the shRNA-encoding lentiviral plasmid using 1 mg/mL polyethyleneimine (Polysciences, Warrington, PA, USA). After 48 h of incubation, the cell supernatant was collected and centrifuged for 3 h at 20,000× *g* at 16 °C. Lentiviral vector pellets were resuspended in Williams’ Media E (Thermo Fisher Scientific, Waltham, MA, USA) and stored at −80 °C until needed. For the titration, a p24 enzyme-linked immunosorbent assay (Cell Biolabs, San Diego, CA, USA) was performed.

### 4.4. Generation of imPTEC Lines

Three different PTEC lines were produced by transduction of 80% confluent primary PTEC monolayers with *SV40* large T-ag encoding lentiviral vector carrying the gene sequence of mCherry as a reporter together with 8 mg/mL protamine sulfate (Sigma-Aldrich) at 37 °C overnight. After 24 h, mCherry expressing PTECs were sorted using a FACS Aria Fusion cell sorter (BD Biosciences). PTECs expressing the mCherry reporter gene were collected and expanded for further assays, while non-transduced cells were used as a control. mCherry-positive PTEC purity was evaluated by fluorescence microscopy using Keyence BZ-8100E microscope (KEYENCE). Likewise, flow cytometry analysis was performed using an SA3800 Spectral Analyzer (Sony, Tokyo, Japan) by detecting mCherry fluorescence.

### 4.5. Evaluation of Immortalization Efficiency

*SV40* large T-ag expression levels were analyzed at primary cells (*n* = 3) at passages 0, 5, and 10 and imPTECs (*n* = 3) at passages 0, 5, 10, 15, and 20. Total RNA was isolated using an RNeasy Mini Kit (Qiagen, Hilden, Germany) and reverse transcribed to cDNA with a High-Capacity cDNA Reverse Transcription Kit (Applied Biosystems, Foster City, CA, USA). Quantitative real-time polymerase chain reaction (qRT-PCR) was performed using specific *SV40* large T-ag primers (Fw: 5′-ACT CTT GCT TGC TTT GCT ATT TAC ACC AC-3′, Rv: 5′-TGT ATA GCA GTG CAG CTT TTT C-3′, and *SV40* large T-ag probe 5′-ACT CTT GCT TGC TTT GCT ATT TAC ACC AC-3′) (TIB Molbiol Syntheselabor GmbH, Berlin, Germany).

### 4.6. Assessment of Proliferation and Cell Cycle Analysis

To determine the population proliferation rate, PTECs were passaged (ratio 1:2) once they reached confluence. This assay was performed in 12-well plates using CnT-Prime medium. Passages were counted for up to 40 days. To calculate the cell cycle state of primary and imPTECs, 3 PTEC lines at low passages (passage 2) and at high passages (passage 10) were examined. Cell cycle analysis was performed using Vybrant™ DyeCycle™ Violet Stain (Thermo Fisher Scientific) following the manufacturer’s protocol. Therefore, cells were stained for 1 h and analyzed by flow cytometry immediately after incubation. 

### 4.7. Albumin Uptake Assay by Endocytosis

The ability of primary and imPTECs to uptake albumin via endocytosis was evaluated by the incubation of confluent monolayers in 24-well plates with 50 µg/mL bovine serum albumin (BSA)-FITC (Sigma-Aldrich) for 1 h at 37 °C. After incubation, uptake was arrested by washing PTECs with ice-cold PBS. Cells were detached by using TrypLE^TM^ Express Enzyme (Gibco) and analyzed by flow cytometry using a BD FACSCanto™ II Clinical Flow Cytometer System (BD Biosciences). Cells incubated without BSA-FITC served as a control.

### 4.8. Migration Assay

Migration assay was performed with primary and imPTECs at passage 2 or passage 10. PTECs were seeded on 24-well flat-bottom plates and cultured in CnT-Prime medium. After 6 h, a scratch (~300 µm) was performed. Afterwards, PTECs were cultured for 48 h, and pictures were taken every 12 h using a Lumascope 620 microscope (Etaluma, San Diego, CA, USA). Images were analyzed using Aperio ImageScope Software v12.4.0.5043 (Leica Biosystems, Deer Park, IL, USA). 

### 4.9. Assessment of Silencing SLA Class I and Class II Expression 

SLA class I and II downregulation was achieved by transduction with lentiviral vectors containing shRNA sequences targeting porcine β2m and CIITA. To evaluate the SLA class I and class II silencing effect, non-transduced, non-silenced (shNS), and silenced (shβ2m + shCIITA) PTECs were analyzed. The silencing effect was evaluated at mRNA levels by qRT-PCR and at protein levels by flow cytometry after stimulation with interferon-gamma (IFN-γ) (50 ng/mL) for 48 h. 

Total RNA was isolated from three SLA-silenced PTEC lines and reversed transcribed using a High-Capacity cDNA Reverse Transcription Kit (Applied Biosystems). qRT-PCR was utilized to detect β2m, CIITA, and SLA-DRα transcript level expression using Taqman assays (Ss03391156_m1; Ss06941905_g1; Ss03389945_m1; all Thermo Fisher Scientific), respectively. GAPDH (Ss03375629_u1, Thermo Fisher Scientific) expression was used as an endogenous control. 

SLA class I and class II protein expression was determined by flow cytometry. Cell staining was performed using an anti-SLA class I antibody (JM1E3; Bio-Rad Laboratories GmbH, Hercules, CA, USA) and anti-SLA class II DQ antibody (K274.3G8; Bio-Rad Laboratories GmbH) followed by APC-conjugated anti-mouse IgG1 (RMG1-1; Invitrogen) or AlexaFluor488-conjugated goat anti-mouse IgG secondary antibody (Invitrogen) staining. 

### 4.10. T-Cell Cytotoxicity Assay

Human peripheral blood mononuclear cells (PBMCs) were isolated from 4 healthy donors by density gradient centrifugation using Lymphosep (c.c.pro GmbH, Oberdorla, Germany). T cells (effector cells) were isolated using magnetic-activated cell sorting of human Pan T cell isolation Kit (Miltenyi Biotec, Auburn, AL, USA). Effector cells were co-cultured with non-transduced cells, non-silenced (shNS), and SLA-silenced (shβ2m + shCIITA) PTECs for 40 h days in RPMI 1640 medium (Lonza, Basel, Switzerland) supplemented with 5% human serum AB (c.c.pro GmbH) and IL-2 (100 U/mL) in a ratio of 1:5 (target: effector cells). PTEC (target cells) proliferation was continuously monitored using the xCELLigence Real-Time Cell Analyzer (Agilent Technologies, Santa Clara, CA, USA). Microtiter plates (E-Plates©) (Agilent Technologies) were utilized to measure non-invasive electrical impedance. Cell index values were recorded every 4 h until the experiment was terminated.

After 40 h, the supernatant was collected, centrifuged at 1500 rpm for 5 min, and stored at −80 °C until analysis. Granulocyte–macrophage colony-stimulating factor (GM-CSF), interferon-gamma (IFN-γ), monocyte chemoattractant protein 1 (MCP-1), interleukin 6 (IL-6), interleukin 8 (IL-8), and interferon gamma-induced protein 10 (IP-10) levels were determined using a MILLIPLEX Human Cytokine/Chemokine/Growth Factor Panel A Magnetic Bead Panel (Merck Millipore, Burlington, MA, USA) and a Luminex^®^ 100/200 analyzer (Luminex Corporation, Austin, TX, USA). According to the manufacturer’s instructions, standards and samples were prepared, and cytokine concentrations were calculated by Xponent software version 3.1 (Luminex Corporation).

### 4.11. NK Cell Degranulation Assay

Human NK cells were isolated from three healthy donors. Briefly, PBMCs were isolated by density gradient centrifugation using Lymphosep (c.c.pro GmbH) and NK cells were collected using human NK cell isolation Kit (Miltenyi Biotec). For NK cell degranulation analysis, non-stimulated or 48 h IFN-γ-stimulated (50 ng/mL), non-transduced, non-silenced (shNS), and SLA-silenced (shβ2m + shCIITA) imPTECs were analyzed. Target cells were co-cultured with NK cells for 4 h at 37 °C incubators in a ratio of 1:5 (target: effector). After incubation time, NK cell populations were analyzed using FITC-conjugated anti-CD3 (UCHT1; BioLegend) and AlexaFluor647-conjugated anti-CD56 (B159; BD Biosciences) antibodies. NK cell degranulation was evaluated by detecting the expression of CD107a using a PE/Cy7-conjugated anti-CD107a antibody (H4A3; BioLegend).

### 4.12. Statistical Analyses

All data are reported as mean ± standard deviation. For comparison between two groups, Student’s *t*-test was used. One-way ANOVA with multiple comparisons was applied to compare data with one variable between more than two groups. Two-way ANOVA was used for comparisons of data with two categorical variables between more than two groups. *p*-values of <0.05 were considered statistically significant and defined as * *p* < 0.05, ** *p* < 0.01, *** *p* < 0.001, and **** *p* < 0.0001. All statistical analyses were conducted using GraphPad Prism version 8 software (GraphPad Software Inc, San Diego, CA, USA).

## Data Availability

All data will be provided upon reasonable request to the corresponding author.
